# Pooled peptides from HER-2/neu-overexpressing primary ovarian tumours induce CTL with potent antitumour responses *in vitro* and *in vivo*

**DOI:** 10.1038/sj.bjc.6602259

**Published:** 2004-12-07

**Authors:** A D Gritzapis, S A Perez, C N Baxevanis, M Papamichail

**Affiliations:** 1 Cancer Immunology and Immunotherapy Center, Saint Savas Cancer Hospital, Athens, Greece

**Keywords:** tumour lysates, HER-2/neu, CTL, SCID mice

## Abstract

Unfractionated peptides (MW: up to 10 kDa), derived from HLA-A2.1 positive (+) HER-2/neu-overexpressing primary tumour cell acid cell extracts (ACE), were successfully used to generate *in vitro* cytotoxic T lymphocytes (CTL). Primary tumour cells were collected from peritoneal malignant effusions of patients with ovarian cancer. Acid cell extracts-induced CTL specifically lysed in an HLA-A2-restricted manner HER-2/neu^+^ autologous primary tumour cells as well as HER-2/neu^+^ tumour cell lines. In addition, adoptive transfer of such CTL significantly prolonged the survival of SCID mice xenografted with HLA-A2.1^+^, HER-2/neu^+^ human breast and ovarian tumour cell lines. Acid cell extracts collected from HLA-A2.1^+^ HER-2/neu negative (−) primary ovarian tumours induced HLA-A2.1-restricted CTL with weak *in vitro* and *in vivo* antitumour capacity, suggesting that HER-2/neu peptides within ACE from HER-2/neu-overexpressing primary ovarian tumour cells are immunodominant. The results presented herein serve as a rationale for the initiation of vaccination studies in patients with HER-2/neu-overexpressing ovarian tumours utilising autologous tumour-derived ACE.

It is now well established that tumours growing *in vivo* provide their antigens to the immune system either as soluble proteins by shedding from the surface of viable cells or as ‘apoptotic bodies’ from dead cells. Tumour antigens, during the process of ‘crosspriming’ are taken up by antigen-presenting cells (APC) which present them to cytotoxic T lymphocytes (CTL), thereby eliciting the induction of antitumour immune responses (Carbone *et al*, 1998). A significant number of tumour antigens, which function as CTL targets, have now been identified using various approaches including molecular genetic techniques, immunoaffinity purification of MHC molecules followed by acid elution of peptides and subsequent sequence determination, serological analysis of recombinant cDNA expression libraries, and identification of peptide sequences with MHC class I-binding motifs through computer algorithms (Wang and Rosenberg, 1999).

The use of defined tumour antigens in peptide-based vaccination studies for cancer immunotherapy is advantageous because it provides pure antigenic preparation that enhances the effectiveness of the vaccine and at the same time minimises the possibility of adverse autoimmune reactions due to the absence of irrelevant material such as self-proteins shared between tumour cells and healthy tissue. However, the use of defined CTL epitopes in peptide-based vaccines provides several obstacles to effective cancer immunotherapy. Firstly, it is not always certain that a tumour epitope eliciting CTL responses *in vitro* can also be recognised by CTL *in vivo*, thereby eliciting antitumour responses (Anichini *et al*, 1996; Kirkin *et al*, 1999). Secondly, tumour peptide-specific CTL efficiently recognising peptide-pulsed target cells do not necessarily recognise tumour cells presenting naturally processed epitopes (Van Elsas *et al*, 1995; Zaks and Rosenberg, 1998). Finally, peptide-based vaccinations may induce the generation of tumour escape variants lacking the expression of a particular peptide epitope (Pawelec, 1999).

An alternative approach that circumvents these problems is the use of unfractionated peptides, as a source of tumour antigens, isolated from tumour cell lysates produced upon acid treatment (ACE) (Nair *et al*, 1997). The presence of multiple peptides within ACE may ensure the induction of several peptide-specific CTL clones of different affinities for a given peptide which will synergistically mount an effective antitumour response. The existence of a plethora of tumour peptide-specific CTL clones will reduce the risk of generation of tumour escape variants since tumour cells will be attacked via the recognition of multiple antigens restricted by several HLA alleles (Pawelec, 1999). In addition, peptide mixtures present within ACE circumvent the need for characterising tumour-specific antigens and open the possibility for vaccination protocols in cases where tumour antigens have not been identified. Simultaneous presentation of CTL and T_H_ epitopes by dendritic cells (DC) may be highly increased when tumour lysates are used as sources of antigenic material (Baxevanis *et al*, 2000).

The potency of unfractionated peptides within tumour cell lysates as tumour vaccines has been explored in preclinical studies (Nair *et al*, 1997; Fields *et al*, 1998; Gatza and Okada, 2002; Schnurr *et al*, 2002; Wen *et al*, 2002; Gad *et al*, 2003; Graner *et al*, 2003; Vegh and Mazumder, 2003) as well as in clinical trials for the immunotherapy of melanoma (Nestle *et al*, 1998) and renal cell carcinoma (Holth *et al*, 2002). Their majority of these experimentations included DC as the preferred APC. Given the technical difficulties in obtaining high numbers of pure mature DC from both bone marrow cells and peripheral blood monocytes, the vaccination protocol were limited to only restricted numbers of injections of lysate-loaded DC per patient (Nestle *et al*, 1998; Holth *et al*, 2002). This may have caused incomplete clinical responses, which could possibly have been improved by increasing the number of injections, provided of course enough numbers of DC were available. Another point that was not thoroughly explored in these studies considers the actual effector cell type that mediated the antitumour response. *In vivo* depletion studies in animal models indicated that most of this effect was mediated by CD8^+^ cells (Fields *et al*, 1998; Gatza and Okada, 2002) not discriminating between T or NK cells expressing this marker. Concerning the clinical studies, mostly DTH responses to the keyhole limpet haemocyanin, used as helper antigen, were measured. *Ex vivo* CTL responses to certain tumour-associated peptides were weakly positive and most important it was not checked whether such CTL could lyse patients’ tumour cells.

In this study, we used for the first time unfractionated peptides isolated by acid treatment from lysates of HER-2/neu-overexpressing, HLA-A2.1^+^ primary ovarian tumour cells to generate *in vitro* autologous antitumour CTL. Our data point to the conclusion that HER-2/neu peptides within ACE from HER-2/neu-overexpressing ovarian primary tumour cells are immunodominant and that such preparations can be used in the cellular adoptive immunotherapy of ovarian cancer.

## MATERIALS AND METHODS

### Patients

HLA-A2.1^+^ patients (*n*=5) with histologically confirmed ovarian cancer (clinical stage III and IV, tumour grade III) were enrolled in this study. Patients fulfilled the following criteria: Karnofsky performance status >80%; bilirubin levels <1.7 ng dl^−1^ and creatinine levels <2.2 ng dl^−1^; leucocyte count >3.000 *μ*l^−1^ and platelet count >100 000 *μ*l^−1^. They had not received any antineoplastic therapy during the 3 week preceding the onset of the study. All patients were apprised of the study, and consents were obtained consistent with the policies of St Savas Cancer Hospital.

### Preparation of effusion cells

Specimens of peritoneal effusions (1–2 l) from patients, collected during routine aspirations, were spun at 400 *g* for 5 min to sediment cells. Malignant effusion-associated mononuclear cells (MEAMNC) and tumour cells were isolated from the cell pellet as previously described (Baxevanis *et al*, 1994). In agreement with our previous report (Baxevanis *et al*, 2000), freshly isolated MEAMNC consisted mainly of T cells (>68% CD3^+^ cells) with almost equal numbers of CD3^+^CD8^+^ (28–37%) and CD3^+^CD4^+^ (32–40%) T cells. A substantial number of monocytes (CD14^+^ cells) was also detected (10–16%). In all cases examined, the number of MEAMNC isolated from the effusions ranged from 380 to 1.090 × 10^6^ and that of tumour cells from 170 to 650 × 10^6^. Both MEAMNC (as effectors) and tumour cells (as targets) were utilised in experiments when viability was >80%.

### Monocyte isolation and generation of DC

CD14+ cells were isolated from total MEAMNC using the Monocyte Isolation Kit (Miltenyi Biotec), comprising a mixture of CD3, CD7, CD19, CD45RA, CD56, and anti-IgE Abs coupled to MACS Microbeads according to the manufacturer's constructions. Dendritic cells were generated from monocytes in the presence of 800 U ml^−1^ rGM-CSF (Shering-Plough, Brinny, Innishannon, Ireland) and 500 U ml^−1^ rIL-4 (R&D Systems Europe, Abington, UK), as described (Baxevanis *et al*, 2000). The percentage of DC recorded was >50%, as tested on the expression of CD3^−^, CD14^−^, CD16^−^, CD20^−^, CD40^−^, CD80^−^, CD83^−^ and MHC class II+ phenotype. In all cases, the number of DCs generated from CD14+ cells ranged from 20 to 50 × 10^6^.

### Immunophenotyping of tumour cells

HER-2/neu expression was determined on single tumour cells isolated from the malignant effusions by flow cytometry, using the PE-conjugated anti-HER-2/neu mAb (clone Neu 24.7; Becton Dickinson, Mountain View, CA, USA). The expression of HER-2/neu was qualified by comparing the mean fluorescence intensity (MFI) of the primary tumour cells with the MFI of tumour cell lines expressing HER-2/neu at different levels (i.e. HER-2/neu expression of the MDA-231 cell line is scored as 1 (negligible expression), of MCF-7 as 2 (intermediate expression) and of SKBR-3 as 3 (overexpression)) (Sotiropoulou *et al*, 2003a). In the five patients examined, HER-2/neu expression on primary tumour cells from the peritoneal effusions was scored as 3 (Ova-1, Ova-2, Ova-3) or 0 (Ova-4, Ova-5). All tumour cells expressed HLA-A2.1 (% range of expression: 55–65) as determined via use of the BB7.2 mAb (kindly provided by Prof H Rammensee at the Department of Immunology, University of Tuebingen) but were negative for MHC class II gene products. HLA-A2.1 was the only matching allele between patients’ primary tumour cells and the human tumour cell lines used as targets.

### Preparation of ACE

This was performed as described (Baxevanis *et al*, 2000). In brief, an estimate of 1–2 × 10^8^ tumour cells was washed in HBSS (Life Technologies, Gaithersburg, MD, USA), followed by homogenisation in 1 ml homogenisation buffer. Eluates from cells were titrated with 10% trifluoroacetic acid and clarified by two successive centrifugations at 2500 *g* and 80 000 *g* for 30 min and 5 h, respectively. Peptides were processed immediately on a Sep-Pak C18 cartridge (Waters, Bedford, MA, USA) equilibrated prior to use with 3 ml acetonitrile, followed by 3 ml deionised water. The eluate was allowed to flow through the cartridge by gravity, the column was washed with deionised water and bound material was finally eluted with 2 ml 60% acetonitrile in deionised water and lyophilised in a Speed-Vac (Heto Lab Equipment, Allerod, Denmark). Dry product was reconstituted in HBSS and further processed on a Centricon centrifuge concentrator (Amicon, Beverly, MA USA) with a cutoff of 10 kDa by centrifugation at 2500 *g* at 4°C for 2–3 h. The filtrate was aliquoted and stored at −20°C.

### Peptide synthesis

Peptides were synthesised by the solid-phase method with an Ecosyn P peptide synthesiser (Eppendorf-Biotronik, Hamburg, Germany) using the Fmoc strategy and a 4-carboxybenzyl alcohol resin. Purification was performed by high-performance liquid chromatography. The purity was >95%. The following HER-2/neu-derived peptides were synthesised: HER-2/neu (9_665_), HER-2 (9_689_), HER-2 (9_369_), HER-2 (10_952_), HER-2 (9_851_) and HER-2 (9_402_). These are high binding affinity peptides for HLA-A2.1, eliciting strong CTL activity *in vitro* (Fisk *et al*, 1995; Rongcun *et al*, 1999; Baxevanis *et al*, 2002). The gp100-derived peptide gp (9_154_) and the MART-1/Melan-A-derived peptide Melan (9_27_) were used as controls. The latter peptides have been demonstrated to elicit *in vitro* HLA-A2.1 CTL-restricted activity (Kirkin *et al*, 1999).

### Pulsing with ACE or HER-2/neu-derived peptides

The amount of ACE for pulsing APC (DC or MEAMNC) or target cells (T2 cells) was determined from its capacity to induce maximal stabilisation of HLA-A2.1 expression on T2 cells (Fisk *et al*, 1995). Based on estimations from our previous report (Baxevanis *et al*, 2000) and the present one, we pulsed 1 × 10^6^ DC or MEAMNC with ACE extracted from 5 × 10^6^ primary ovarian tumour cells for stimulating autologous responder MEAMNC. When T2 cells were used as targets, loading was performed by incubating 1 × 10^6^ cells either with ACE from 5 × 10^6^ tumour cells or with the indicated HER-2/neu synthetic peptides at a 20 *μ*g ml^−1^ final concentration. Incubations with ACE or peptides were performed overnight in CO_2_ incubators.

### Generation of ACE-specific CTL lines *in vitro*

Responder MEAMNC (1 × 10^6^ cells ml^−1^) were cultured in 24-well plates (Costar, Cambridge, MA, USA) with 1 × 10^5^ ml^−1^ irradiated (3000 rad) autologous DC pulsed with ACE, derived from the autologous primary tumour cells, in a total volume of 2 ml X-VIVO 15 medium supplemented with 1% autologous serum (=complete medium), rIL-7 (20 ng ml) (R&D Systems Europe, Abington, UK) and 25 IU ml^−1^ rIL-2 (Cetus, Emeryville, CA, USA) in CO_2_ incubators. After 5–7 days, one-half of the medium was replenished with fresh medium containing 40 ng ml^−1^ rIL-7 and 50 IU ml^−1^ rIL-2. After an additional 5 days incubation (=stimulation phase), recovered responders were washed and restimulated with thawed autologous irradiated (3000 rad) ACE-pulsed MEAMNC used as APC at a cell ratio of 1 : 2. Fresh rIL-2 (25 IU ml^−1^) was also added to the medium. After two additional rounds of restimulation, as above, (=restimulation phase) bulk MEAMNC effectors were tested in the *in vitro* cytotoxicity assays. Before transfer to SCID mice, the same effectors were expanded in tissue culture with anti-CD3 mAb (Biosciences; clone SK7) according to a method previously reported (Riddell *et al*, 1996).

### Cytotoxicity assay

The cytotoxicity assay was performed as previously described (Baxevanis *et al*, 1994). Briefly, effector CTL MEAMNC (1 × 10^6^ ml^−1^) were placed in 100 *μ*l aliquots into wells of 96-well V-bottomed plates (Costar). As targets, primary tumour cells or tumour cell lines were labelled with sodium [^51^Cr] chromate (Radiochemical Centre, Amersham, UK; 100–200 *μ*Ci isotope per 1–2 × 10^6^ target cells) and added to effectors at the indicated E : T ratios. For peptide recognition, T2 cells were incubated overnight at 26°C together with 20 *μ*g ml^−1^ peptide (or ACE), washed and then labelled. Incubation was performed for 6 h in CO_2_ incubators. In some experiments, blocking with an anti-HLA-A2.1 mAb was performed by preincubating those target cells with 10 *μ*g ml^−1^ of the BB7.2 mAb. Cytotoxicity values were considered to indicate significant recognition of a target when the differences between mean values (from triplicate analyses) for percent lysis of the particular target (e.g. pulsed T2 cells, primary tumour cells or transfected tumour cell lines) and unloaded T2 cells or HLA-A2^−^ tumour targets were ⩾10% at an E : T ratio of 40 : 1 and statistically significant (*P*<0.05).

### Quantitation of cytokines in culture supernatants

An ELISA kit specific for IL-2 was obtained from R&D Systems Europe. IFN-*γ* was quantitated with an ELISA kit from Endogen (Boston, MA, USA). Assays were performed according to the manufacturer's instructions.

### Tumour rejection models

Groups of 10 SCID mice were inoculated s.c. with 5 × 10^5^ cells of each tumour cell line in 0.5 ml PBS. Injections with ACE-specific CTLs (2 × 10^7^ cells in 0.5 ml PBS) were administered intraperitoneally (i.p.) at the time point when tumour was palpable (ca. 10–16 days after inoculation of mice with the tumour cell lines). Tumour size was monitored regularly every 4 days and was expressed as the product of the perpendicular diameters of individual tumours. Each animal experiment was repeated at least twice. The observation was terminated with the euthanasia of mice when the tumour mass grew up to 200–250 mm^2^ in diameter. A nonparametric Wilcoxon rank test was used in the statistical analysis of the size of the tumour in individual groups. The difference was considered statistically significant when *P*<0.05.

## RESULTS

### ACE-induced CTL with cytotoxic activity against HER-2/neu+ HLA-A2.1^+^ tumour cell lines and primary autologous tumour cells

**Figure 1:**
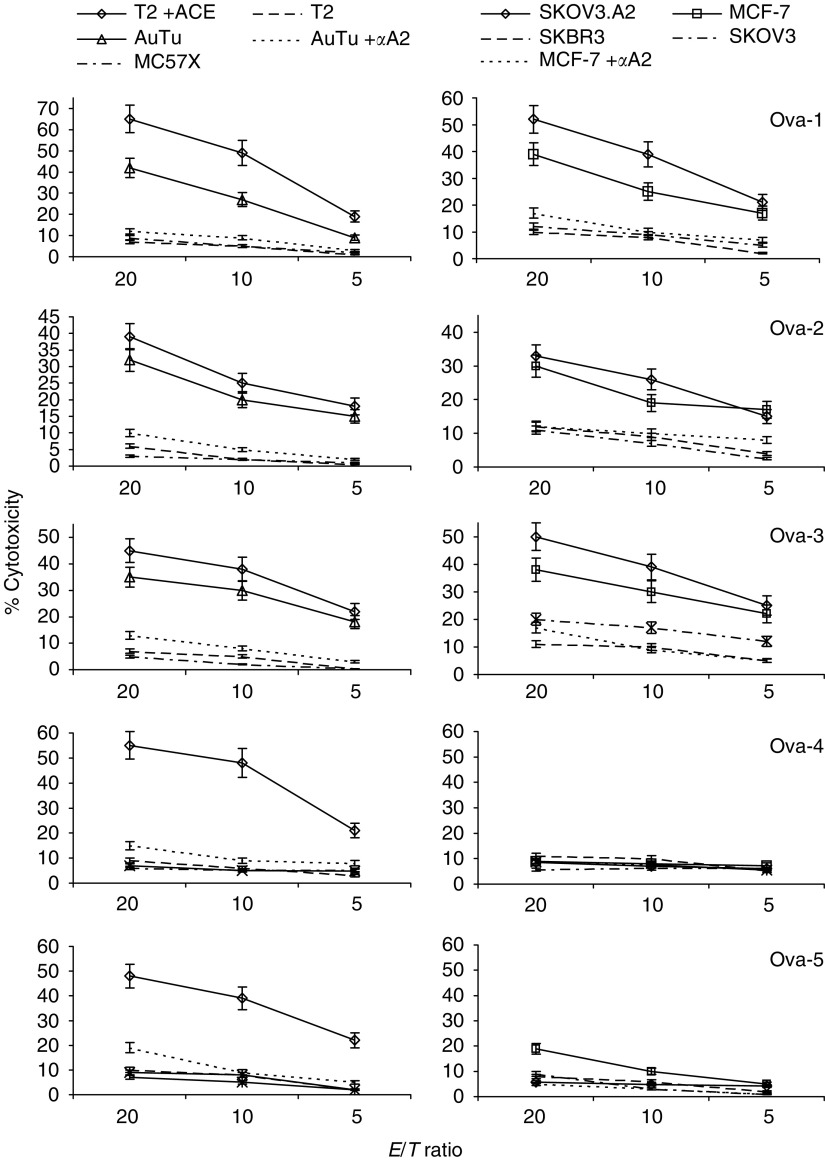
Cytotoxic responses mediated by MEAMNC bulk CTL after *in vitro* culture with ACE derived from HLA-A2.1 primary ovarian tumour cells overexpressing HER-2/neu (Ova-1, Ova-2 and Ova-3) or being HER-2/neu^−^ (Ova-4 and Ova-5). Cytotoxicity was tested against various targets also including the autologous primary tumour cells (AuTu) from which the ACE was extracted. In some cases, anti-HLA-A2.1 mAb (*α*A2) was added throughout the cytotoxicity assay. One experiment of three performed is shown. Mean values±s.d. from triplicate cultures are shown.

Acid cell extracts prepared from HER-2/neu-overexpressing, HLA-A2.1^+^ primary tumour cells were tested for their ability to elicit specific CTL from autologous MEAMNC. These tumour cells were isolated from peritoneal effusions of three patients with ovarian cancer (designated Ova-1, Ova-2, Ova-3). Dendritic cells differentiated from monocytes within the MEAMNC population were used, pulsed with ACE, as APC. After a stimulation phase in the presence of IL-2 and IL-7, which was followed by three restimulations with autologous MEAMNC pulsed with ACE, as APC, the bulk effector MEAMNC population consisted of both CD8^+^ and CD4^+^ T cells (% mean±s.d. in the three bulk cultures: 54±10 and 43±10, respectively). Although total MEAMNC effectors were utilised in the cytotoxicity experiments (see below), for simplicity reasons these are referred to as CTL. Bulk CLT cultures efficiently lysed T2 targets pulsed with ACE derived from the autologous ovarian primary tumour cells (Figure 1, left panel). The same cultures were also tested for CTL activity against various targets including the autologous ovarian primary tumour cells and HLA-A2.1^+^, HER-2/neu^+^ tumour cell lines. As shown in Figure 1, bulk CTL activity induced by ACE from Ova-1, Ova-2 and Ova-3 primary tumour cells could be demonstrated against the respective autologous tumour cells (Figure 1, left panel) as well as against the HER-2/neu^+^ ovarian cell line SKOV3 transfected to express HLA-A2.1 (SKOV3.A2; provided by Dr C-G Ioannides, Department of Gynecologic and Oncology and Immunology, university of Texas, MD Anderson Cancer Center) and the HER-2/neu^+^ HLA-A2.1^+^ breast cancer cell line MCF-7 (Figure 1, right panel). In contrast, the same effectors failed to lyse the HER-2/neu-overexpressing but HLA-A2^−^ parental SKOV3 and breast cancer cell line SKBR3 (HLA-A2.1 was the only matching allele between the primary ovarian tumours and these tumour cell lines) or the HER-2/neu^−^ mouse fibrosarcoma cell line MC57X(H-2^b^). Cytotoxicity was HLA-A2.1-restricted since it was to a great extent inhibited in the presence of BB7.2 mAb (% range of inhibition against the autologous tumour and MCF-7 targets: 56–77 at a cell ratio of 40 : 1) (Figure 1). The finding that our ACE-induced CTL specifically lysed HER-2/neu^+^ HLA-A2.1^+^ tumour cells demonstrates their ability to recognise naturally processed peptides expressed and presented on the tumour cell surface in the context of HLA-A2.1 molecules.

In contrast to ACE prepared from the Ova-1, Ova-2 and Ova-3 tumours, ACE derived from the HER-2/neu^−^ HLA-A2.1^+^ Ova-4 and Ova-5 primary tumours induced only a modest CTL activity against the autologous primary tumour targets or the HER-2/neu^+^ HLA-A2.1^+^ SKOV3.A2 and MCF-7 tumour lines (Figure 1). Such cytotoxic responses were apparently directed against ACE peptides other than those derived from HER-2/neu, which were also presented in the context of HLA-A2.1 molecules, since (i) there was significant inhibition of the response in the presence of BB7.2 mAb (range of % inhibition for the autologous tumours, SKOV3.A2 and MCF-7 targets: 54–79); (ii) only marginal killing was observed against the HLA-A2.1^−^ SKOV3 and SKBR3 cell lines (Figure 1, right panel) and (iii) significant levels of cytotoxicity (55% and 48%) were observed when ACE-pulsed T2 cells were used as targets (Figure 1, left panel).

The relatively high percentages of cytotoxicity against the ACE-pulsed T2 targets *vs* the weak lysis of the autologous tumour targets as well as the SKOV3.A2 and MCF-7 cell targets suggests that the peptides within ACE prepared from HER-2/neu^−^ ovarian primary tumour cells Ova-4 and Ova-5 are to a great extent not naturally expressed on the surface of the tumours and therefore cannot be considered as strongly immunogenic. The percentages of CD4^+^ T cells within the bulk MEAMNC population by culture termination were at relatively equal levels (33 and 47%), with those in bulk cultures sensitised by ACE from the HER-2/neu^+^ primary tumours Ova-1, Ova-2 and Ova-3, thus excluding the possibility that low numbers of CD4^+^ T cells, apparently providing insufficient help for CD8^+^ CTL, could account for the low cytotoxic responses against the autologous primary tumour cells or the tumour cell lines.

### HER-2/neu peptides recognised by ACE-induced MEAMNC bulk cultures

To more precisely delineate the immunodominant role of HER-2/neu-derived peptides within the ACE from HER-2/neu-overexpressing ovarian primary tumour cells, we attempted to link the cytotoxic activity mediated by the ACE-induced CTL against the HER-2/neu^+^ HLA-A2.1^+^ tumour targets with their ability to lyse T2 cells pulsed with HER-2/neu synthetic peptides known to represent HLA-A2.1-restricted CTL epitopes. As presented in Table 1, effectors induced with ACE from Ova-1 tumours recognised peptides HER-2 (9_851_), HER-2 (9_435_), HER-2 (9_665_) and HER-2 (9_369_). Peptides HER-2 (9_435_) and HER-2 (9_369_) were also recognised by CTL bulk cultures induced with ACE from Ova-2 and Ova-3 primary tumours. The same effectors lysed T2 cells pulsed with HER-2 (9_689_) but not the same targets pulsed with peptides HER-2 (9_851_) or HER-2 (9_665_). None of the effectors recognised HER-2 (10_952_) and HER-2 (9_402_) or control peptides Melan A (9_27_) and gp (9_154_). In addition, there was no reactivity by any of the three CTL cultures against. As expected, CTL bulk cultures sensitised with ACE from the HER-2/neu^−^ Ova-4 and Ova-5 tumours did not recognise any of the HER-2/neu peptides tested.

### Cytokine production by the bulk MEAMNC effectors

**Figure 2:**
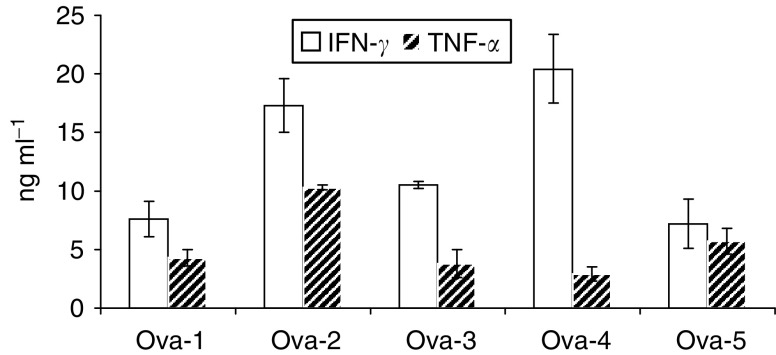
Cytokine production by the MEAMNC bulk CTL. Cytokine determinations were performed in parallel with the cytotoxicity assays. Cytokines were quantitated in culture supernatants after the end of the restimulation phase. Ova-1 through Ova-5 indicates the primary ovarian tumour cells which were used as source for ACE preparation. Mean values±s.d. from three parallel cultures (from the same MEAMNC donor) are shown.

Parallel experiments were set up to quantitate cytokine production by the same ACE-induced MEAMNC effectors used in the cytotoxicity experiments. Cytokine levels were measured in culture supernantants by culture termination (i.e. after the third restimulation). With the use of commercially available ELISA kits, both IFN-*γ* and TNF-*α* were detected at varying concentrations. The data from Figure 2 clearly show that IFN-*γ* and TNF-*α* levels produced by the MEAMNC bulk population sensitised with ACE from the HER-2/neu^−^ Ova-4 and Ova-5 primary tumours (in ng ml^−1^: 20.4 and 7.2 for IFN-*γ*; 2.9 and 5.7 for TNF-*α*, respectively) were almost within the range of concentrations quantitated in the supernatants of MEAMNC cultures stimulated by ACE from the HER-2/neu^+^ Ova-1, Ova-2 and Ova-3 primary tumours (range: 7.6–17.3 for IFN-*γ* and 1.3–10.5 for TNF-*α*). Only marginal levels of IL-4 could be detected in all five cases (<0.1 ng ml^−1^; data not shown). These data demonstrated that the culture conditions used favour the production of Th1 cytokines supporting antitumour immunity. In addition, they support the idea that the low cytotoxicity induced by ACE preparations from Ova-4 and Ova-5 HER-2/neu^−^ primary tumours should rather be attributed to an inadequate recognition of tumour targets than to qualitative differences in cytokine secretion profiles by the MEAMNC effectors.

### ACE-induced MEAMNC effectors when adoptively transferred mediate antitumour responses in xenografted SCID mice

**Figure 3:**
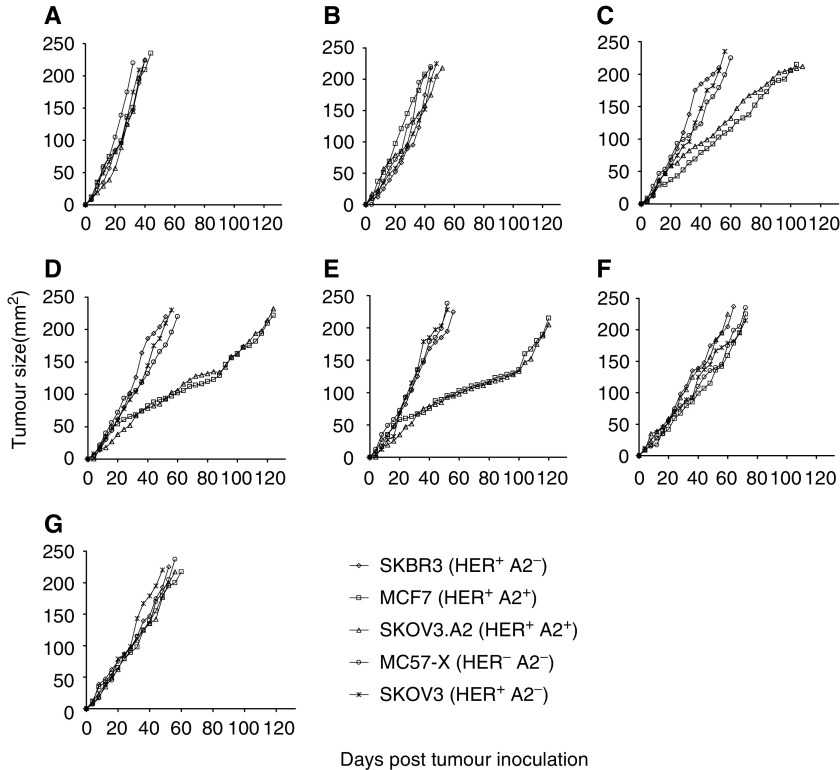
Adoptive transfer of MEAMNC bulk CTL induced by ACE from HER-2/neu-overexpressing HLA-A2.1^+^ primary ovarian tumour cells (Ova-1, Ova-2 and Ova-3) suppress the growth of human HER-2/neu^+^, HLA-A2.1^+^ tumour lines in SCID mice (**C**, **D** and **E**, respectively). (**F**, **G**) Results with MEAMNC bulk CTL induced by ACE from HER-2/neu^−^ HLA-A2.1+ primary Ova-4 and Ova-5 tumour cells, respectively. Cytotoxic T lymphocytes were i.p. injected at 2 × 10^7^ cells per injection (one injection was given per mouse) in SCID mice with s.c. growing tumours which were induced upon inoculation, 10–16 days before, with the indicated human tumour lines or the mouse fibrosarcoma MC57X. Control CTL (**B**) consisted of CTL specific for the melanoma gp (9_154_) peptide. (**A**) Growth of tumour lines in untreated SCID mice. Cytotoxic T lymphocytes used for adoptive transfer were expanded (as described in Materials and Methods) from the same cultures shown in the previous figures and presented in Table 1. One experiment out of two with similar results is shown. In rapidly growing tumours, the s.d. was too low (i.e. <5% of the means) and thus omitted.

Next, we attempted to determine whether our ACE-induced CTL effectors could exert antitumour effects *in vivo* by protecting SCID mice against the growth of the human tumour cell lines, which were previously used as targets in the *in vitro* cytotoxicity experiments. Cytotoxic T lymphocytes effectors after the restimulation phase were expanded in the presence of anti-CD3 mAb and rIL-2 and transferred at 2 × 10^7^ cells per mouse to SCID mice with palpable subcutaneous tumours (these mice had been inoculated 10–16 days before with the tumour cell lines). As shown in Figure 3A, these tumour lines were growing relatively fast and formed large tumours (>200 mm^2^ area) within 30–44 days in untreated animals. Such vigorous tumour growth was not restrained when mice were treated with a human CTL line specific for the HLA-A2.1-restricted epitope gp (9_154_) of the melanoma-associated gp100 protein (Figure 3B). This line was capable of lysing human melanoma lines expressing both HLA-A2.1 and gp100 (data not shown). Transfer of the HLA-A2.1-restricted and HER-2/neu-recognising MEAMNC effectors sensitised with ACE from Ova-1 (Figure 3C), Ova-2 (Figure 3D) or Ova-3 (Figure 3E) induced a significant delay in the growth of SKOV3.A2 and MCF-7 tumour lines (growth of both tumours reached an area >200 mm^2^ 104–138 days after inoculation; *P*<0.01 compared to nontreated mice or mice treated with the gp (9_154_)-specific CTL). The same effectors remained without any effect when SCID mice were inoculated with the HER-2/neu^+^ HLA-A2.1^−^ tumour lines SKBR3 or SKOV3 or with the murine MC57X fibrosarcoma (Figure 3C–E), demonstrating the specificity of the *in vivo* responses. In agreement with the *in vitro* results (Figure 1), transfer of MEAMNC effectors induced *in vitro* with ACE from the HER-2/neu^−^ primary tumours did not exert any significant antitumour effects *in vivo* (Figure 3F and G).

## DISCUSSION

In this study, we demonstrate for the first time the capacity of ACE from HER-2/neu-overexpressing primary ovarian tumour cells to induce *in vitro* autologous CTL from MEAMNC with the ability to exert *in vivo* antitumour effects against HER-2/neu^+^ human tumour cell lines. Such CTL recognised peptides within ACE which were naturally processed and expressed on the surface of tumour cells in the context of the HLA-A2.1 allele. This was indicated by the following findings: first, ACE peptides induced stabilisation of HLA-A2.1 molecules on T2 cells and such ACE-pulsed T2 cells were efficiently lysed by our ACE-induced CTL effectors; second, HER-2/neu^+^, HLA-A2.1^+^ primary tumour cells as well as human tumour cell lines of breast and ovarian origin were lysed in an HLA-A2.1-restricted fashion since the cytotoxicity was to a great extent abrogated in the presence of the mAb BB7.2, and third, HER-2/neu-overexpressing but HLA-A2.1^−^ human cell lines were not lysed.

By pulsing T2 cells with synthetic HER-2/neu CTL peptides, we could identify immunodominant nanomers that were recognised by our ACE-induced bulk CTL. These included peptides HER-2 (9_369_), HER-2 (9_435_) (both recognised by bulk CTL induced by ACE preparations from Ova-1, Ova-2 and Ova-3 primary HER-2/neu-overexpressing tumours), peptide HER-2 (9_689_), which may be recognised by Ova-2 and Ova-3 induced CTL, and peptides HER-2 (9_851_) and HER-2 (9_665_), which were recognised by one of the three bulk CTL. These HER-2/neu peptides have been demonstrated to bind to HLA-A2.1 molecules with high affinities (Fisk *et al* 1995; Rongcun *et al*, 1999), thereby eliciting CTL activity among tumour-associated lymphocytes in patients with breast and ovarian cancer (Fisk *et al*, 1995; Rongcun *et al*, 1999; Baxevanis *et al*, 2002). Moreover, we have recently demonstrated the capacity of these peptides to sensitise CTL for lysing their autologous tumour cells in patients with prostate, lung and colorectal cancer (Sotiropoulou *et al*, 2003a, 2003b). Thus, our data demonstrate the utility of ACE preparations as polyepitope carriers. Such carriers besides the HER-2/neu peptides identified, most likely contain others which remain to be identified by screening the entire HER-2/neu protein for sequences with HLA-A2.1-binding motifs, followed by peptide synthesis, loading onto T2 targets and testing in cytotoxicity assays with ACE-induced CTL effectors.

Although HLA-A2.1 is the most popular allele for presenting HER-2/neu CTL epitopes, there are also other MHC class I alleles functioning as restriction elements for HER-2/neu peptide presentations (Kiessling *et al*, 2002). Accordingly, recognition of the HER-2/neu^+^, HLA-A2.1^−^ SKOV3 or SKBR3 cell lines by our ACE-induced bulk CTL effectors could also be accomplished through HLA- class I alleles other than HLA-A2.1. However, according to the serotyping, only the HLA-A2.1 subtype was shared between the primary HER-2/neu-overexpressing Ova-1, Ova-2 and Ova-3 tumours (which served as source for the ACE preparation) and the SKOV3 or SKBR3 tumour cell lines and therefore only HER-2/neu peptide-specific and HLA-A2.1-restricted CTL clones would have a chance to recognise them. Of course, recognition could also have been established through CTL clones specific for HLA-A2.1-restricted peptides other than those derived from the HER-2/neu oncoprotein. Although this is quite likely, still the contribution of such clones would not add much to overall killing of the HLA-A2.1^+^ tumour targets. This hypothesis is based on our findings demonstrating that CTL induced by ACE from the Ova-4 and Ova-5 HLA-A2.1^+^ but HER-2/neu^−^ primary tumours only weakly lysed their autologous tumour targets (i.e. the Ova-4 and Ova-5 primary tumours) as well as the HLA-A2.1^+^ and HER-2/neu^+^ SKOV3.A2 and MCF-7 tumour cell lines. Thus, it seems possible that peptides within ACE from the HER-2/neu^−^ primary ovarian tumours are weakly immunogenic most likely due to inefficient expression and presentation on the tumour cell surface. This is supported by the findings that both the Ova-4- and the Ova-5-ACE induced CTL, although exerting weak cytotoxic activity against the HLA-A2.1^+^ HER-2/neu^−^ autologous primary tumours and against the HLA-A2.1^+^ HER-2/neu^+^ tumour cell lines, still were capable of (i) efficiently lysing T2 targets pulsed with the same ACE (i.e. those prepared from Ova-4 and Ova-5 primary tumours), and (ii) producing substantial amounts of IFN-*γ* and TNF-*α* after the restimulation phase. Both parameters were comparable to those of CTL induced by ACE from the HER-2/neu-overexpressing Ova-1, Ova-2 and Ova-3 primary tumours.

SCID mice were protected against inoculation with HER-2/neu^+^, HLA-A2.1^+^ human tumour cell lines when bulk CTL specific for ACE prepared from the HER-2/neu-overexpressing primary ovarian tumours were adoptively transferred. Independently of the HER-2/neu peptides recognised, Ova-1CTL (recognising HER-2 (9_851_), HER-2 (9_435_), HER-2 (9_665_) and HER-2 (9_369_)), Ova-2CTL (recognising HER-2 (9_689_), HER-2 (9_435_) and HER-2 (9_369_)) and Ova-3CTL (recognising HER-2 (9_689_), HER-2 (9_435_) and HER-2 (9_369_)) induced similar levels of protection demonstrating the immunodominance of these HER-2/neu epitopes, but not excluding, however, the possibility that also CTL clones specific for other HER-2/neu epitopes may be included in these bulk CTL populations.

There are several models described so far in the literature utilising different vehicles for vaccination studies with polyepitope constructs. These include attenuated virus vectors (Toes *et al*, 1997; Schneider *et al*, 1998) naked DNA (Thomson *et al*, 1998) or transfected DC (Condon *et al*, 1996). Acid cell extracts preparations from primary tumour cells may be advantageous over such polyepitope vaccines not solely due to the fact that these apparently contain a plethora of CTL epitopes, but also due to the simplicity of the method used for their preparation and administration. In addition, ACE derived from primary tumours may contain T helper epitopes as well (Baxevanis *et al*, 2000), which will contribute to the enhancement of CTL-mediated antitumour responses. In contrast to other reports utilising tumour cell lysates or unfractionated tumour peptides in active immunisation studies (Nair *et al*, 1997; Fields *et al*, 1998; Gatza and Okada, 2002; Schnurr *et al*, 2002; Wen *et al*, 2002; Gad *et al*, 2003; Graner *et al*, 2003; Vegh and Mazumder, 2003), our data emphasise the role of such preparations in the cellular adoptive immunotherapy of cancer by sensitising *in vitro* the effector CTL population.

Collectively, the studies presented herein provide evidence that pooled peptides from HER-2/neu-overexpressing ovarian tumours can be utilised in cellular adoptive immunotherapy of patients with HER-2/neu^+^ ovarian cancer. They also imply that vaccination with such multiepitope preparations may allow enhanced efficacy in the clinical treatment of ovarian cancer. Moreover, the identification of immunodominant HER-2/neu peptides within these unfractionated peptide extracts may help in the collection of peptides to be included in multipeptide vaccines.

## Figures and Tables

**Table 1:** ACE-induced CTL from patients with ovarian cancer recognise HER-2/neu peptides

			**HER-2/neu peptides**						
**Effectors^a^**	**Melan (9_27_)**	**Gp (9_154_)**	**(9_851_)**	**(10_952_)**	**(9_689_)**	**(9_402_)**	**(9_435_)**	**(9_665_)**	**(9_369_)**
Ova-1	7.0^b^	8.5	**21.5^c^**	7.6	11.3	8.8	**47.6**	**38.7**	**39.5**
Ova-2	2.5	6.3	8.5	7.2	**41.7**	12.1	**52.7**	10.3	**35.9**
Ova-3	9.0	5.5	6.9	3.9	**25.6**	3.9	**52.8**	6.7	**30.6**
Ova-4	6.5	7.2	10.2	8.3	7.2	10.5	9.3	7.5	3.9
Ova-5	10.2	3.2	5.6	2.5	5.7	9.6	11.2	6.8	7.5

a MEAMNC sensitised *in vitro* against ACE from autologous HER-2/neu-overexpressing primary tumour cells (Ova-1–Ova-5) were tested as effectors for cytotoxicity against T2 cells pulsed with the indicated HER-2/neu peptides. Effector to target ratio=40 : 1.

b Indicates mean % cytotoxicity values from triplicate cultures. The s.d. (not shown) was always less then 15% of the mean values.

c Statistically significant cytotoxic responses (*P*<0.01) over background responses (i.e. cytotoxicity against unpulsed T2 targets) and over cytotoxicity against control Melan (9_27_) and gp (9_154_) peptides. Bold values indicate significance in comparison with control values.
